# The medical diagnostic approaches with phylogenetic analysis for rare Brucella spp. diagnosis in Taiwan

**DOI:** 10.7603/s40681-015-0009-6

**Published:** 2015-06-06

**Authors:** Ni Tien, Yun-Ju Sung, Yi-Chih Chang, Bang-Jau You, Michelle Liang, Yun-Ping Lim, Wen-Yu Ho, Hsiu-Shen Lin, Mao-Wang Ho, Chen-Mao Ho, Chao-Chin Chang, Yu-Ching Lan

**Affiliations:** 1 Department of Laboratory Medicine, China Medical University Hospital, 404, Taichung, Taiwan; 2 Department of Medical Laboratory Science and Biotechnology, China Medical University, 404 Taichung, Taiwan; 3 Department of Chinese Pharmaceutical Sciences and Chinese Medicine Resources, China Medical University, 404 Taichung, Taiwan; 4 Department of Microbiology, UCLA, CA 90095 Los Angeles, USA; 5 Department of Pharmacy, College of Pharmacy, China Medical University, 404 Taichung, Taiwan; 6 Department of Laboratory Medicine, China Medical University Beigang Hospital, 651 Taichung, Taiwan; 7 Department of Internal Medicine, School of Medicine, China Medical University, 404 Taichung, Taiwan; 8 Graduate Institute of Microbiology and Public Health, National Chung Hsing University, 402 Taichung, Taiwan; 9 Department of Health Risk Management, China Medical University, No. 91, Hsueh-Shih Road, 404 Taichung, Taiwan

**Keywords:** Brucella, Traditional biochemical methods, 6s *rRNA*, Phylogenetic analysis, Anti-microbial, susceptibility test

## Abstract

Brucellosis is a bacterial zoonotic disease which can be easy to misdiagnose in clinical microbiology laboratories. In the present study, we have tried to improve the current clinical method for detecting *Brucella* spp. and its antibiotic characteristics. Our method begins with detecting the clinical isolate through traditional biochemical methods and automatic identification systems. Then, we move on to editing the sequence for BLAST allows us to compare 16s *rRNA* sequences with sequences from other species, allowing the gene level to be determined. Next, the phylogenetic analysis of multiple genetic loci is able to determine the evolutionary relationships between our bacteria strain and those from other locations. Finally, an anti-microbial susceptibility test hones in on the level of antibacterial activity that the bacteria displays. Employing these four steps in concert is extremely effective in identifying rare bacteria. Thus, when attempting to determine the identity of rare bacteria such as *Brucella*, utilizing these four steps from our research should be highly effective and ultimately prevent further identification errors and misdiagnoses. The standards we have suggested to identify rare bacteria strains is applicable not only to *Brucella*, but also to other rarely encountered bacteria.

## 1. Introduction

Brucellosis is one of the most common zoonotic diseases, with more than 500,000 new cases yearly. Its prevalence is more than 10/100,000 population in some endemic areas such as France, Israel, and most of Latin America, the Middle East, northern Africa, and central Asia [1, 2]. The disease is transmitted by consumption of unpasteurized dairy products or by occupational contact with infected animals. In the past 15 years, the epidemiology of human brucellosis has increasingly evolved through tourism and cases of animal brucellosis [2]. Furthermore, infected objects are the most common cause of laboratory-transmitted infections in laboratory workers [3, 4]. *Brucella* spp. has been classified in the high risk group of pathogens [5].

Since *Brucella* spp are intracellular bacteria, relapse is often seen [6-9]. The features of *Brucella* spp include being a facultative intracellular pathogen, lacking capsules, flagellates, endosperms or native plasmids, and being slow growing and small (0.5-0.7 × 0.6-1.5 µm) gram-negative coccobacilli (GNCB). Brucellosis usually causes systemic diseases in the osteoarticular, hematological, hepatobiliary, gastrointestinal, cardiovascular, and central nervous systems [10]. Common clinical symptoms of brucellosis are characterized by high fever, myalgia, and arthralgia of the large joints. Apart from these main symptoms, brucellosis can also mimic various multisystem diseases by exhibiting wide clinical polymorphism and nonspecific symptoms, which frequently lead to misdiagnosis and treatment delay [11, 12]. Brucellosis may be difficult to diagnose because of its wide clinical polymorphism. Previous identification experiences have had problems with errors. Laboratories had been report some cases of Brucella initially misdiagnosed by automatic identification systems before. These errors can lead to misdiagnosis, delayed treatment, and ultimately, the infection of even more individuals [11-14].

Since 1980, Taiwan has been free of this disease after an eradication program was implemented [15-17]. However, in 2011 a few cases were reported. These cases were traced back to North Africa and Malaysia [16-18]. They indicate that the pathogen can still pose a threat to public health in Taiwan despite previously being eradicated. There are worries that the overall capacity of Taiwanese physicians may be lacking due to inexperience in identification of the bacteria and subsequent misdiagnoses in clinical microbiology laboratories.

Therefore, the aim of this report is to share our experiences, to culture and identify the findings of this rare pathogen in Taiwan, and to compare the phylogenetic relevance of our genetic sequence with other epidemic strains in the geographical areas mentioned above. This research will aid in refining our understanding about the source of pathogens, thus allowing clinical microbiology laboratory workers to pay more attention to the identification and diagnosis of the rare *Brucella* spp. [19].

## 2. Materials and methods

In this study, we detected the clinical isolate by utilizing traditional biochemical methods and automatic identification systems which include the BD Phoenix system and API 20E and 32 GN identification kits. Furthermore, we used the 16s *rRNA* sequences method for determining gene level. We performed an antibiotic sensitivity test. In addition, we also carried out phylogenetic analysis. By analyzing our strain of bacteria and comparing it with those from other geographical areas, we were able to determine the evolutionary relationship between the strain from Taiwan and other areas’ strains.

### 2.1. Collection and identification of bacteria isolate

The conventional biochemical tests used included Oxidase-positive, urease-positive, H_2_S production, dye tolerance such as basic fuchsin and thionin and sero-agglutination tests. We routinely employed the BD Phoenix NMIC/ID-2 commercial kit (Becton Dickinson diagnostic System, Sparkes, MD, USA). Inoculation was performed according to the manufacturer’s instructions. The API 20E and 32 GN systems (Biomerieux SA, Marcy l’Etoile, France) were also used to identify the strain. Inoculation, reading, and interpretation of panels were performed according to the manufacturer’s instructions [20].

### 2.2. 16S ribosomal RNA gene sequencing

Sampling and sample preparation: The bacteria from positive blood culture specimens of the patient were plated on Trypticase soy agar with 5% defibrinated sheep blood (BBL Microbiology Systems, Cockeysville, Md.) and incubated aerobically for 2 days at 37°C. Several visible colonies were selected and suspended in 600 µl TE buffer and adjusted to MacFaland 3.0 cell density for nucleic acid extraction.

Nucleic acid extraction: DNA was extracted from fluid samples (600 µl) using the Genomic DNA Mini Kit (Geneaid, Taiwan). The appropriate protocols were followed according to the manufacturer’s instructions; with a final elution volume of 50 µL. Extracted DNA was stored at 4°C until required for PCR.

Amplification of 16s *rRNA* genes: The 16s *rRNA* gene from the microorganisms was amplified by PCR. A primer pair conconsisting of 8f (5’-GAGAGTTTGATCCTGGCTCAG-3’) and 1492r (5’-TACGGCTACCTTGTTACGACT-3’) [21, 22] was used to amplify nearly 1500-bp fragments of the 16s *rRNA* genes. The samples were amplified in the following PCR mixture: 10 µmol of each primer in a 2X buffer containing 4 mM MgCl_2_, 0.4 mM of each deoxynucleoside triphosphate, 0.05 U *Taq* DNA polymerase, and 40 mM (NH_4_)_2_SO_4_ (Ampliqon, Skovlunde, Denmark) in a final volume of 50 µL. The following temperature cycles were used: 94°C for 5 min, 30 cycles of 94°C for 1 min, 55°C for 1 min, and 72°C for 1 min and 30 s, and a final extension at 72°C for 7 min. All reactions were conducted in a GeneAmp 9700 thermocycler (Applied Biosystems, Foster City, Calif.).

16s *rRNA* gene sequencing and alignment: Sequencing primers were chosen from a pair of previously described oligonucleotides, 8f and 1492r. Sequencing was performed with a 3730xl DNA Analyzer (Applied Biosystems, Foster City, Calif.). Sequences were aligned using the BioEdit suite of programs (www.mbio.ncsu.edu/BioEdit/bioedit.html), and the identity was evaluated by checking against existing sequences using BLAST (http://www.ncbi.nlm.nih.gov/BLAST). Sequencing of the 16s *rRNA* gene fragments showed a clear division of sequences into *Brucella melitensis* [22-24].

### 2.3. The multiple genetic loci for phylogenetic relationship identification

A previous study already successfully determined the sequences of multiple genetic loci in order to examine the relationships between *Brucella* isolates [25]. In order to further identify the genetic relationship among *Brucella* strains in this study and others strains in the GenBank, we extracted the *Brucella* DNA and amplified multiple genetic loci of the isolate, including *aroA, glK, danK*, and *gyrB* partial gene fragments (Table 1) for phylogenetic analysis [25].

### 2.4. Polymerase chain reactions (PCRs) and sequencing

Four distinct genome fragments were amplified by PCR using the primers shown in Table 1. PCR reaction mixes were prepared for each sample by mixing 10 µmol of each primer in a 2X buffer containing 4 mM MgCl_2_, 0.4 mM of each deoxynucleoside triphosphate, 0.05 U Taq DNA polymerase, and 40 mM (NH_4_)_2_SO_4_ (Ampliqon, Skovlunde, Denmark) in a final volume of 30 µL. Cycling parameters were as follows: 94°C for 5 min. followed by 30 cycles of 94°C for 1 min., 53°C for 1 min. and 72°C for 1 min., and a final polishing step of 72°C for 10 min. Products were separated by agarose gel electrophoresis to check for efficiency of amplification and to ensure that only a single product of the expected size was present. The DNA products were sequenced by using a GeneAmp 9700 thermocycler (Applied Biosystems, Foster City, Calif.).

### 2.5. Phylogenetic anlaysis

The *aroA, glK, dank* and *gyrB* gene partial segments sequence data were edited using Bioedit for alignment, and then these data were combined with each other and before undergoing phylogenetic analysis. Sequences of the four loci were concatenated to produce a 1675 bp sequence for each genotype sequence. Phylogenetic analysis was performed with the MEGA software, Version 3.1. The neighbor joining tree was constructed with the concatenated sequence data of the four loci (1,675 bp) using the neighbor joining approach. The Jukes-Cantor model, which is based on the assumption that all nucleotide substitutions are equally likely, was used to determine genetic distances. The percentage bootstrap confidence levels of internal branches were calculated from 1,000 resamplings of the original data.

**Table 1:** Oligonucleotide sequences used for the amplification and sequencing of four genetic loci.

Locus	Function	Primer sequences	Length (bp)
*aroA*	3-phosphoshikimate 1-carboxyvinyltransferase	5’ GACCATCGACGTGCCGGG 3’ 5’ YCATCAKGCCCATGAATTC 3’	565
*glK*	glucokinase	5’ TATGGAAMAGATCGGCGG 3’ 5’ GGGCCTTGTCCTCGAAGG 3’	475
*danK,*	chaperone protein	5’ CGTCTGGTCGAATATCTGG 3’ 5’ GCGTTTCAATGCCGAGCGA 3’	470
*gyrB*	DNA gyrase B subunit	5’ ATGATTTCATCCGATCAGGT 3’ 5’ CTGTGCCGTTGCATTGTC 3’	469

### 2.6. Antimicrobial susceptibility test of *Brucella* isolate from clinical specimens

The antibiotic susceptibility test applied the paper disc diffusion method and the minimal inhibition concentration test; MIC. Tigecycline (TGC) MICs were determined by the E test (Biomerieux, Sweden). Mueller-Hinton agar supplemented with 5% sheep’s blood agar plate (Oxoid, UK) was inoculated with bacterial suspensions with a equivalent to a 0.5 McFarland turbidityand was interpreted 2 days after incubation in ambient air. The susceptibility testing of tetracycline (Te) (30 µg/ml), streptomycin (STR) (300, 10 µg/ml), rifampin (RIF) (5 µg/ml), and trimethoprim-sulfamethoxazole (TMP-SMZ) (1.25/23.75 µg/ml) was determined by disk diffusion method. Mueller-Hinton agar supplemented with 5% sheep’s blood was inoculated with suspensions of bacteria with equivalent 0.5 McFarland turbidity and was interpreted 48 h after incubation in ambient air.

## 3. Results

### 3.1. Conventional identification

The gram- negative coccobacilli on the BAP plate are batter growth and appeared small and white. The glossy quality of the batter growth suggests that the gram-negative coccobacilli were of the smooth-quality type. The size of the bacteria was about 0.5-0.8 µm × 0.6-1.5 µm. There were no colonies growing on the EMB plate. The conventional biochemical tests showed a positive reaction that included the catalase, oxidase, and urease. Automated instruments and the API system (32 GN and 20 E) were employed to identify the bacteria. The results presented two species, *Ochrobactrum antropi* and *Myrodes* spp., respectively. The Phoenix instrument gave an *Ochrobactrum antropi* result, and the species was identified with 90% confidence. The API 20E identification was analyzed, and then code number 0210004 presented Myroides / Chryseobacterium indologenes (% id: 47.4, T = 0.92), Bordetella / Alcaligenes / Moraxella spp. (% id: 25.7, T = 0.87) and *Ochrobactrum antropi* (% id: 21.7, T = 0.82). The API 32 GN identification code number 00000000002 was *Myroides* spp. (% id: 90.0, T = 1.00). 

### 3.2. 16S ribosomal RNA gene sequencing

The 16s *rRNA* gene sequences were aligned by the BioEdit program and BLAST. A 99% similarity between *Brucella melitensis* and *Brucella ovis* was discovered. The 16s *rRNA* gene sequence illustrated a homology between these two species. We further conducted phylogenetic analysis from multiple genetic loci in order to examine the relationships between *Brucella* isolates.

### 3.3. The phylogenetic relationships with other *Brucella*

To recognize the phylogenetic relationships of this *Brucella* strain, sequences of the four loci were concatenated to produce a 1675 bp sequence. The multiple genetic loci that were analyzed included *aroA, glK, dank* and *gyrB* partial gene fragments. The reference sequences with whole genomes came from the Gen- Bank. The topology of the phylogenetic reference tree from the four loci was similar to the tree from the whole genome. The percentage bootstrap confidence levels of internal branches were calculated from 1,000 re-samplings of the original data. After comparison with the brucellosis in the Genbank as reference sequences, the bacterial strain in this study was clustered with the *Brucella melitensis* strains in a significantly monophyletic branch of the neighboring joining tree. The branch lengths represent the genetic variation between Taiwan *Brucella melitensis* and strains from other geographic areas.

### 3.4. Antimicrobial susceptibility test

**Fig. 1:**
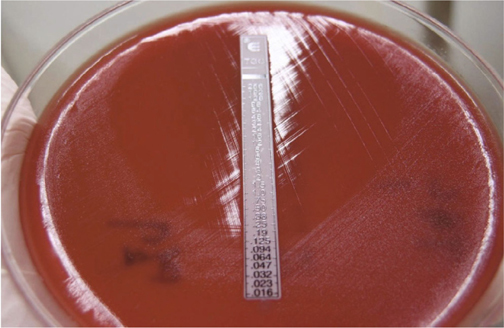
Tigecycline MICs results were determined by the E test.

In the tigecycline MICs, results were determined by the E test with turbidity between 0.5, 0.75, and 1.0 McFarland, but the results appeared all the same as 0.094 µg/ml (Figure 1). The inhibitory zone size of tetracycline (Te), streptomycin (STR), rifampin (RIF) and trimethoprim-sulfamethoxazole (TMP-SMZ) are shown in Table 2.

### 4. Discussion

Brucellosis has become a rare disease in developed and developing countries. As the infectious dose is very low, infections are an occupational risk for farmers, veterinarians, abattoir workers, laboratory personnel, and others who work with animals and consume their products [18, 26]. The increase in business and leisure travel to brucellosis-endemic countries has led to the importation of the disease into non-endemic areas [26]. Two problems arise from this importation. First, clinicians in non-endemic areas often have insufficient experience with brucellosis and have difficult making the correct diagnosis. Second, there could be failures in notifying laboratories, and brucellosis bacteria could be misidentified by using commercial automatic diagnosis system. These two problems are serious enough to deserve special attention in Taiwanese clinical microbiology laboratories [27].

The “gold standard” in the diagnosis of brucellosis is bacterial isolation, which requires long cultivation periods and is often unsuccessful. Because of this, we back-tracked the identification process and found that *Brucella* spp. is not included regularly in the database of automatic identification systems. The results of our tests presented *Ochrobactrum antropi* and *Myroides* / *Chryseobacterium indologenesthe*, respectively, and were given by two identification systems, the Phoenix and the API 20E systems. The confirmed rates were untrustworthy [9, 12, 13]. Whereas *Brucella* spp. are classified as highly pathogenic biosafety level 3 agents, only two species of the genus *Ochrobactrum* (*O. anthropi* and *O. intermedium*) have been associated with opportunistic immunocompromised human disease [28]. It can be seen that errors in identification of *Brucella* spp. may not only affect physicians’ treatments, they may also affect the safety of laboratory personnel.

*Ochrobactrum* represent a distinct genus distantly related to *Achromobacter* but phylogenetically closely related to the *rRNA* superfamily IV of the *Alphaproteobacteria*—in particular, to *Brucella* and *Phyllobacterium* [9]. The close relationship to *Brucella* was emphasized in 1998 by Velasco *et al*. [29]. This close relationship has led to misidentification of *Brucella melitensis* as *Ochrobactrum anthropi* in the past. In previous research, the 16s *rRNA* sequences of *Brucella* spp. and *O. intermedium* were found to be 98.6% identical [29]. Like the previous research group report [14], our study also had a similar misclassification experience with *Brucella* spp. in our automatic identification and 16s nucleotides blast. The result of our 16s *rRNA* BLAST illustrated a homology between *Brucella melitensis* and *Brucela ovis*.

Results that are not accurate have the very real potential of misleading the examiner(s) of these bacteria. One could potentially send the wrong bacteria culture report and cause clinicians to misdiagnose [12, 13]. There is currently a growing trend in errors due to the increasing utilization of automated equipment for microbial identification. Limitations in the instruments’ databases and inability to distinguish similar phenotype strains mean laboratory staff must sometimes use traditional options such as characterizing bacterial colonies and examining staining patterns as well as biochemical reactions to determine what bacteria are in a given sample. However, a clinical microbiology laboratory can also think ahead and identify bacteria and bacterial genotypes using molecular biological techniques. 16s *rRNA* analysis methods and phylogenetic analysis can provide more accurate reports in order to make up for the inability of automated systems to distinguish between closely related bacterial strains.

**Table 2 –:** Susceptibility testing results were determined via disk diffusion method.

Antibiotics	Concentration (µg/ml)	Inhibitory zone (mm)
Tetracycline	30	28
Streptomycin	10	30
	300	40
Rifampin	5	21
Trimethoprim-sulfamethoxazole	1.25/23.75	22

Ever since the early microbiological work performed by Wilson (30), researchers have been developing increasingly sophisticated methods of classifying *Brucella* species. However, despite technical advances in genotyping, the methods we have chosen have been able to roughly generate the same evolutionary relationships as those seen in whole genome phylogenies, especially in clinical approaches with a short time-span. Multilocus sequence typing trees in our study of *Brucella* roughly approximate the whole-genome phylogeny but use only four housekeeping genes. Although each approach to genotyping has its value, particularly when low-cost genotyping is the goal, only wholegenome sequencing can capture the full extent of genetic variation. Furthermore, only whole-genome phylogenies allow us to gauge the accuracy of previous genetic methods. Understanding the evolutionary framework of the genus *Brucella* is essential for designing assays that differentiate the various strains or biovars, and only by rooting our phylogeny can we understand the directionality of the evolutionary process. A future study might pursue a strategy for tracing the relationship between strains of *Brucella* in Taiwan, China or other neighboring countries.

The CLSI-M100-S20 specification standards of susceptibility of *Brucella* spp. illustrated streptomycin ≦ 8 µg/ml, tetracyclin ≦ 1 µg/ml, doxycycline ≦ 1 µg/ml, gentamicin ≦ 4 µg/ml, trimethoprim-sulfamethoxazole (SXT) ≦ 2/38 µg/ml. When we manipulated the antibiotic sensitivity test, we found that when bacterial growth is slow and the colonies are small, they cannot take advantage of automated identification systems to perform the MIC test. The interpretation of inhibition zone size does not follow the CLSI standards. The same scenario is found in a previous report [11, 12]. Resistance to *Brucella* is not common, but research has pointed out that minimum inhibitory concentration of ceftriaxone and streptomycin (MIC) has been on the rise [7]. Intermediate rifampin susceptibility strains also have become widespread [8]. Kuwait and Mexico have found a good bacteriostatic effect that includes tetracycline, amikacin, gentamicin [11, 12], streptomycin and ciprofloxac for *Brucella* spp. However, rifampin and trimethoprim-sulfamethoxazole’s (SXT) antibacterial effects have been decreasing. The anti-microbial susceptibility test used in previous studies [15, 17] was the disk diffusion method. But the disk diffusion method is an atypical anti-microbial susceptibility test, meaning it is nearly impossible to interpret the results. In our interpretation, we have followed the standard that ≦ 16 mm indicates low anti-Brucella activity and >16 mm is evidence of good anti-*Brucella* activity.

**Fig. 2:**
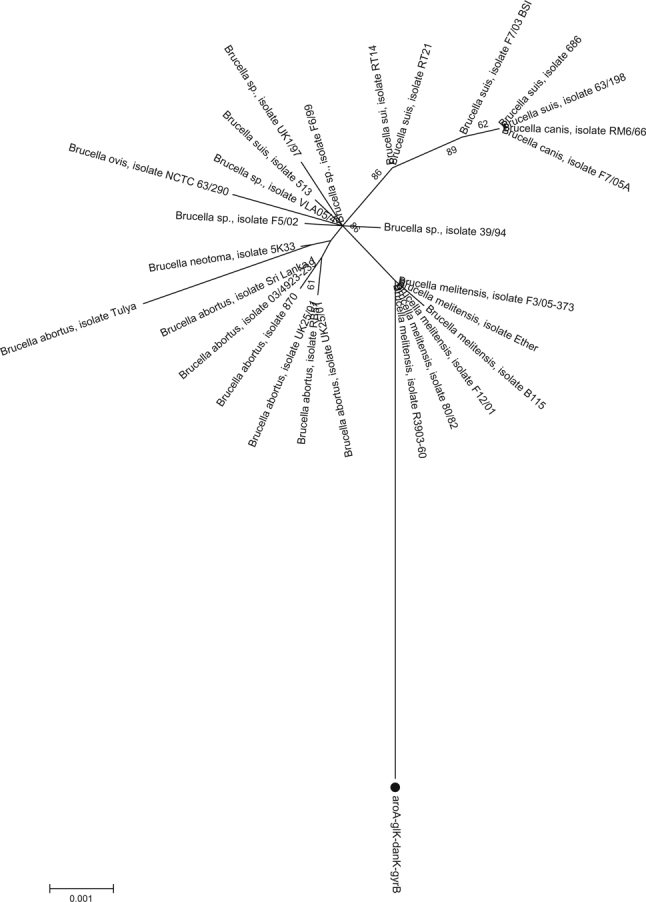
The Neighbour joining tree was constructed with the concatenated sequence data of the four loci (1,675 bp) using the neighbour joining approach. The percentage bootstrap confidence levels of internal branches were calculated from 1,000 resamplings of the original data.

Contrary to cases of brucellosis previously discovered in Taiwan, our report may be the first to suspect cases of local infection. A review of brucellosis infection cases in Taiwan found that the first cases of infection of *B. abortus* occurred in 1978 when university veterinary students came into contact with infected cattle. Later in 1994, a report from 1980-1981 tracked and collected all contacts with infected cattle or other animals by veterinarians, laboratory workers, and farmers, and analyzed if they had been infected with *B. abortus*. Results showed about a 42.1% sero-positive reaction. But these results were never confirmed as being from outside or local cases [22]. However, in 2011 Taiwan also had four cases confirmed from outside the country [23]. In Taiwan it is still possible to become infected by touching infected animals such as deer. The main clinical signs of human Brucellosis are often nonspecific clinical manifestations. Clinicians in Taiwan may easily overlook the possibility of a Brucellosis infection. From this study, our aim is to increase the awareness of laboratory staff as well as aid physicians in their clinical and diagnostic abilities. In addition, we hope to raise awareness about the process of identifying bacteria in clinical microbiology laboratories.

## 5. Conclusion

This is the first study to provide both an improved genotype and phenotype analysis of Taiwan *Brucella* infection in clinical works. Through our research, we built a standard method of four steps for detecting the *Brucella* spp. and its antibiotic characteristics. We have worked to improve the standards by which we identify rare bacteria strains, and to make them applicable not only to *Brucella*, but also to other rarely-encountered bacteria. Our standard method begins with detecting the clinical isolate through traditional biochemical methods and automatic identification systems. The second step is the editing sequence for BLAST that allows one to compare 16s *rRNA* sequences with sequences from other species, allowing the gene level to be determined. In the third step, a phylogenetic analysis of multiple genetic loci is able to determine evolutionary relationships between our bacteria strain and those from other locations. Finally, an anti-microbial sensitivity test hones in on the level of antibacterial activity that the bacteria displays. Employing these four steps in concert is extremely effective in identifying rare bacteria. Thus, when attempting to determine the identity of rare bacteria such as *Brucella*, utilizing these four steps from our research will be highly effective and will hopefully ultimately prevent further identification errors and misdiagnoses.
